# Heritable Genomic Fragment Deletions and Small Indels in the Putative ENGase Gene Induced by CRISPR/Cas9 in Barley

**DOI:** 10.3389/fpls.2017.00540

**Published:** 2017-04-25

**Authors:** Eszter Kapusi, Maria Corcuera-Gómez, Stanislav Melnik, Eva Stoger

**Affiliations:** Department of Applied Genetics and Cell Biology, University of Natural Resources and Life SciencesVienna, Austria

**Keywords:** barley, *Hordeum vulgare*, genome editing, crop, cereals, induced mutation, CRISPR/Cas

## Abstract

Targeted genome editing with the CRISPR/Cas9 system has been used extensively for the selective mutation of plant genes. Here we used CRISPR/Cas9 to disrupt the putative barley (*Hordeum vulgare* cv. “Golden Promise”) endo-*N*-acetyl-β-D-glucosaminidase (ENGase) gene. Five single guide RNAs (sgRNAs) were designed for different target sites in the upstream part of the ENGase coding region. Targeted fragment deletions were induced by co-bombarding selected combinations of sgRNA with wild-type cas9 using separate plasmids, or by co-infection with separate *Agrobacterium tumefaciens* cultures. Genotype screening was carried out in the primary transformants (T0) and their T1 progeny to confirm the presence of site-specific small insertions and deletions (indels) and genomic fragment deletions between pairs of targets. Cas9-induced mutations were observed in 78% of the plants, a higher efficiency than previously reported in barley. Notably, there were differences in performance among the five sgRNAs. The induced indels and fragment deletions were transmitted to the T1 generation, and transgene free (sgRNA:cas9 negative) genome-edited homozygous ENGase knock outs were identified among the T1 progeny. We have therefore demonstrated that mutant barley lines with a disrupted endogenous ENGase and defined fragment deletions can be produced efficiently using the CRISPR/Cas9 system even when this requires co-transformation with multiple plasmids by bombardment or *Agrobacterium*-mediated transformation. We confirm the specificity and heritability of the mutations and the ability to efficiently generate homozygous mutant T1 plants.

## Introduction

Several platforms exist for specific genome editing using designer nucleases, including zinc finger nucleases (ZFNs; Bibikova et al., 2003), transcription activator-like effector nucleases (TALENs; Boch et al., 2009), and the clustered, regularly interspaced, short palindromic repeats (CRISPR) system, often paired with Cas9, the CRISPR-associated protein from *Streptococcus pyogenes* (Barrangou et al., 2007; Brouns et al., 2008; Garneau et al., 2010). Unlike ZFNs and TALENs, which are dimeric nucleases whose target specificity requires DNA–protein interactions, Cas9 is an RNA-guided endonuclease whose specificity depends on the sequence of its single guide RNA (sgRNA). The latter hybridizes to a 20-nt (nucleotide) complementary DNA target (the protospacer), and catalyzes a double-stranded break (DSB) 3–4 bp upstream of the protospacer adjacent motif (PAM), a short and degenerate sequence (5′-NGG-3′ or 5′-NAG-3′ for *S. pyogenes* Cas9) which is required for Cas9 to recognize the protospacer. The DSB induces endogenous repair mechanisms, the resolution of which depends on the repair pathway and the presence or absence of donor DNA: the presence of donor DNA similar to the target region can favor homologous recombination, whereas the absence of homologous donor DNA favors repair by non-homologous end joining (NHEJ), which is error-prone and leads to occasional nucleotide substitutions but usually short insertions and deletions (indels; Pacher and Puchta, 2016). The main benefits of the CRISPR/Cas9 system include its low cost, high efficiency and simplicity (Jinek et al., 2012; Cho et al., 2013; Cong et al., 2013; Hwang et al., 2013; Mali et al., 2013). Whereas different ZFNs and TALENs must be produced for different targets, the same Cas9 enzyme can be used to achieve any modification and only the sgRNA sequence must be changed. However, the sgRNA tolerates a certain number of mismatches so appropriate target sites need to be chosen to minimize unwanted off-target mutations (Pattanayak et al., 2013; Bae et al., 2014).

The CRISPR/Cas9 system has been shown to work in bacteria, yeast, animals, and plants (DiCarlo et al., 2013; Hwang et al., 2013; Jiang et al., 2013a; Bortesi and Fischer, 2015). Many different plants have been modified, including the model organisms *Arabidopsis thaliana* (Feng et al., 2013; Jiang et al., 2013b; Li et al., 2013) and *Nicotiana benthamiana* (Jiang et al., 2013b; Li et al., 2013; Nekrasov et al., 2013; Upadhyay et al., 2013) as well as various crop species, such as wheat (Shan et al., 2013; Upadhyay et al., 2013; Wang Y.P. et al., 2014), sorghum (Jiang et al., 2013b), rice (Feng et al., 2013; Jiang et al., 2013b; Shan et al., 2013; Xie and Yang, 2013), maize (Liang et al., 2014), tomato (Brooks et al., 2014; Ron et al., 2014), potato (Butler et al., 2015), and barley (Lawrenson et al., 2015). The typical outcome of CRISPR/Cas9 editing in crop plants is the introduction of small indels (Jiang et al., 2013b; Zhu et al., 2017). These can be homozygous, heterozygous, or biallelic, and the mutations tend to segregate from the locus expressing the sgRNA and Cas9 nuclease in the T1 progeny (Zhang et al., 2014; Zhou et al., 2014). Lawrenson et al. (2015) induced small indels with the help of Cas9 nuclease in barley and demonstrated transgene-free inheritance of the induced mutations in the T1 and T2 generations, while an off-target mutation was detected in a single plant in the T2 generation. In addition to indels, larger gene fragment deletions have been achieved in *A. thaliana* (Li et al., 2013; Mao et al., 2013), tobacco (Gao et al., 2015), tomato (Brooks et al., 2014), and wheat cell suspension cultures (Upadhyay et al., 2013). Very large chromosomal deletions of 115–245 kb have been induced in rice by simultaneously targeting two distant loci on the same chromosome (Zhou et al., 2014). The removal of small or large chromosomal segments offers the ability to delete exons/protein domains, promoters or even entire genes, which may be preferable to smaller indels that cause frameshift mutations and can leave cells burdened with the synthesis and removal of non-functional polypeptides.

Barley is a model crop species with several advantages, such as its completely sequenced genome (Mayer et al., 2012), true diploidy and well-established genetic transformation methods based on both particle bombardment and *Agrobacterium tumefaciens*. We took advantage of using both methods for barley genetic transformation to find out if there are obvious differences between them when introducing the CRISPR/Cas9 system in this crop. Furthermore, embryogenic barley pollen cultures allow the production of homozygous T1 plants directly from primary transformants (Kapusi et al., 2013). The availability of the barley genome sequence facilitates the design of sgRNAs targeting specific genes for functional studies. Here we set out to create loss-of-function mutations to study the modification of N-glycans in cereal grains. Plants produce two types of N-glycans: oligomannoside-type glycans carrying only core N-acetylglucosamine (GlcNAc) and mannosyl residues, and complex-type glycans also containing other residues such as galactose, xylose, and fucose (Lerouge et al., 1998). N-glycans can be removed by two enzymes: endo-*N*-acetyl-β-D-glucosaminidase (ENGase) and peptide-N(4)-(*N*-acetyl-β-D-glucosaminyl) asparagine amidase (PNGase). ENGase hydrolyses the bond between two GlcNac residues, leaving the peptide chain with the proximal GlcNAc still linked to the asparagine residue, whereas PNGase releases the entire N-glycan and changes the asparagine to an aspartic acid residue. Both activities have been detected in mature barley seeds, and there is a positive correlation between the removal of N-glycans from proteins and the mobilization of storage glycoproteins, reflecting the fact that both ENGase and PNGase activities increase during germination (Vuylsteker et al., 2000). Recombinant glycoproteins produced in cereal grains often carry a single GlcNAc linked to the asparagine residue, possibly reflecting endogenous ENGase activity (Rademacher et al., 2008; Hensel et al., 2015; Vamvaka et al., 2016).

We selected the putative endogenous barley ENGase gene as a candidate for genome editing in order to study the barley machinery for N-glycan modification and removal. This single-copy gene has a high GC content and produces two alternatively spliced mRNAs. The significant advantage of the CRISPR/Cas9 system is that it does not require protein engineering steps, allowing the design and testing of multiple sgRNAs. Accordingly, we tested five different sgRNAs targeting either the sense or antisense DNA strand. We achieved the successful induction of a chromosomal fragment deletion (∼100 bp) between target site pairs and also induced indels that caused frameshift mutations. The mutations were transmitted to the T1 generation and homozygous mutants were identified among the T1 population.

## Materials and Methods

### Construction of Vectors Carrying Cas9 and sgRNA Sequences

**FIGURE 1:**
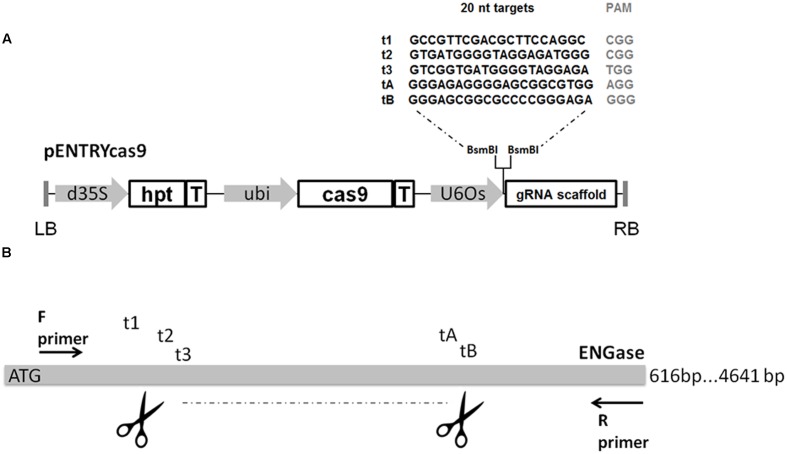
**(A)** Construct design and sgRNA sequences. d35S, double Cauliflower mosaic virus 35S promoter; hpt, hygromycin phoshotransferase; T, terminator; Ubi, maize ubiquitin promoter; U6Os, RNA polymerase III promoter; LB, T-DNA left border sequence; RB, T-DNA right border sequence. **(B)** Design of sgRNA targets for the induction of large deletions.

Five expression vectors were constructed containing DNA sequences representing different sgRNAs (**Figure 1**). The target sequences were integrated into the pcasENTRY vector (DNA Cloning Service, Hamburg, Germany). This vector contains the wild-type cas9 gene under the control of the maize ubiquitin promoter (Christensen and Quail, 1996), the sgRNA scaffold and a hygromycin phosphotransferase (hpt) as a selectable marker. Oligonucleotides complementary to each 20-nt protospacer with appropriate 4-bp overhangs were annealed, phosphorylated, and transferred to the pcasENTRY destination construct using *Bsm*BI to generate unique sgRNAs. This way the target-specific oligos were directly fused to the scaffold of the sgRNA and shuffled into the binary vector carrying the Ubi:cas9 cassette. Each sgRNA insert was sequenced to ensure accuracy using the Tnos-F primer (**Table 1**).

**Table 1:** Forward and reverse primers used in this study.

Primer name	Sequence
ENG-F	5′-GTCTCATCCGCGAGCTCAT-3′
ENG-R	5′-TCCTGTGTTGCAAACATCTCC-3′
Tnos-F	5′-TATGAGATGGGTTTTTATGAT-3′
Cas-F	5′-CTGACGTCGATAAGTTGTTCA-3′
Cas-R	5′-TGATGAACTTGTAGAACTCCT-3′


### Plant Material and Genetic Transformation

Diploid wild-type barley plants (*Hordeum vulgare* cv. “Golden Promise”) were grown in climate chambers under controlled conditions: 14/12°C day/night, 12-h photoperiod and 70% humidity for 10–12 weeks, then 18/16°C day/night, 16-h photoperiod and 70% humidity until maturity. Caryopses were harvested 12–16 days after pollination. Immature embryos, 1–2 mm in size, were used as explants for genetic transformation. The constructs containing the pcas9:sgRNA transfer DNA (T-DNA) were introduced into *A. tumefaciens* strain AGL-1 (Lazo et al., 1991) by electroporation, and overnight cultures were used for transformation as previously described by Hensel and Kumlehn (2004). Cultures of agrobacteria carrying the corresponding pcas9:sgRNA plasmid were mixed in 1:1 ratio and used for immature embryo inoculation. Transgenic barley plants were also produced by particle bombardment (Wan and Lemaux, 1994). Here, the two pcas9:sgRNA constructs were mixed in 1:1 ratio (stock concentration 1 μg/μl), and were co-bombarded after coating onto 0.6 μm gold particles. For each shot 6 μl gold suspension (containing 0.36 mg gold and 1.2 μg of DNA) was applied.

### Molecular Analysis of the Transgenic Barley Plants

Genomic DNA was isolated from barley leaf tissue (Pallotta et al., 2000) for analysis by polymerase chain reaction (PCR). Samples were taken from putative transgenic regenerants approximately 3 months after genetic transformation. At this stage plantlets are 4–6 cm high. The plants were first tested for the presence of the cas9 gene using primers Cas-F and Cas-R (**Table 1**). PCR was carried out using Taq polymerase (Thermo Fisher Scientific, Waltham, MA, USA) in a T100TM thermocycler (Bio-Rad, Munich, Germany). The reaction conditions comprised an initial denaturing step at 95°C for 5 min followed by 35 cycles of 94°C for 30 s, 60°C for 30 s, and 72°C for 1 min. The products were separated by 1% agarose gel electrophoresis. The GC-rich region of the putative barley ENGase gene was amplified using Q5 HiFi high-fidelity DNA polymerase (New England Biolabs, Ipswich, MA, USA) with primers ENG-F and ENG-R (**Table 1**). Each PCR comprised an initial denaturation step at 98°C for 30 s, followed by 32 cycles at 98°C for 10 s, 65°C for 20 s, and 72°C for 20 s, and then a final extension step at 72°C for 2 min. Adenine overhangs were added to each PCR product by incubation with DreamTaq DNA polymerase (Thermo Fisher Scientific, Waltham, MA, USA) for 10 min at 72°C. PCR products were purified by 1.2% agarose gel electrophoresis followed by GeneJet gel extraction (Thermo Fisher Scientific). DNA samples positive for the ENGase fragment were sequenced directly (Microsynth, Balgach, Switzerland) or transferred to the intermediate vector pGEM-T Easy (Promega, Fitchburg, WI, USA). Single colonies were selected by blue/white screening, and the insert was released with *Eco*RI (which does not cut within the target area) and confirmed by 1.2% agarose gel electrophoresis. The amplicon was purified as above and candidate samples were sequenced to characterize the site-specific DNA alterations.

### Genotyping of Primary Transformants (T0) and Their Progeny (T1)

Primary transformants containing the cas9 gene were analyzed to determine whether there were any mutations in the putative barley ENGase gene. In each transformation experiment, two or all five sgRNAs were expressed either by mixing two *A. tumefaciens* strains containing the corresponding pcas9:sgRNA plasmids or by particle bombardment with multiple constructs. The selected target area of the ENGase gene was analyzed to detect Cas9-triggered DSBs. Small mutations were identified by Sanger sequencing and alignment with the wild-type sequence. However, if both target sites were affected by the active nuclease then the resulting larger deletions (∼90 bp) could be detected immediately by PCR. The T1 progeny were similarly analyzed to determine whether the induced mutations were heritable. Genotyping analysis for the heritability of the Cas9 induced mutations was carried out with selected T1 individuals, mainly including primary transgenic plants containing fragment deletions with frameshift mutations. Seeds from selected T0 plants were germinated, genomic DNA was extracted from the leaves, the target area was screened by PCR and Sanger sequencing as above. ENGase edited plantlets were also subject to a PCR reaction using Cas-F and Cas-R primers to determine if the transgene and the induced mutation segregated independently.

## Results

### Experimental Design Using Five Different sgRNAs

As a prerequisite step to produce knock out lines for the putative barley ENGase gene (MLOC_10039.2), its partial sequence of 616 bp was amplified from wild-type barley genomic DNA using primers ENG-F and ENG-R (**Table 1**) and verified by sequencing. Five sgRNA sequences were selected (**Figure 1**) either manually according to certain criteria (guanine was selected as transcriptional start site and high purine content of the 6 nt adjacent to the PAM was favored) in the case of t3 and tB (Hwang et al., 2013; Mali et al., 2013), or using Cas-Designer (http://www.rgenome.net/cas-designer/) in the case of t1, t2, and tA. Each sgRNA targeted a different site in the putative barley ENGase gene, and only t1 was located on the sense strand. All five genomic targets contained an NGG PAM at the 3′ end of the protospacer. In each experiment, more than one (two or all five) target sgRNAs were applied in order to trigger fragment deletions in the selected target area possibly close to the ATG start codon.

**FIGURE 2:**
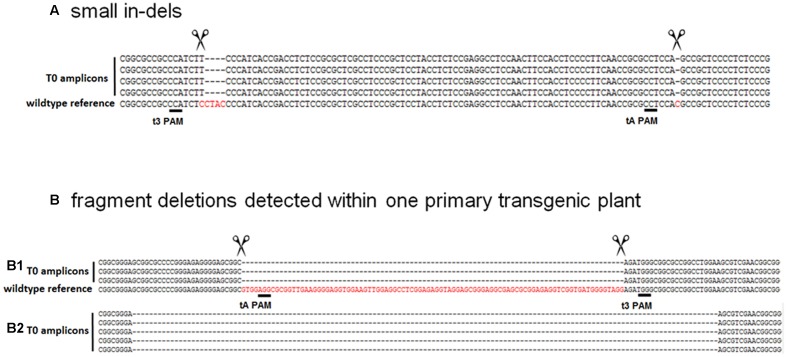
**Typical examples of mutations obtained. (A)** Small indels were commonly found at different target sites. **(B)** Fragment deletions detected within primary transgenic plant B411: panel **(B1)** shows precise DSBs 3–4 nt upstream of the PAM; panel **(B2)** shows DSBs at imprecise sites resulting in more extended deletions. Dashes represent nucleotide deletions.

A total of 32 primary transformants were positive for the cas9 gene (**Table 2**). Eight of these plants were produced by particle bombardment and 24 using *A. tumefaciens*. Mutations in the putative barley ENGase gene were detected in the somatic callus tissue of 25 primary transformants by PCR (**Figure 2** and Supplementary Table S1). Seven of the lines were produced by particle bombardment and 18 lines were produced using *A. tumefaciens*.

**Table 2:** Summarized results after genetic transformation of barley with the cas9:sgRNA constructs.

Sg RNAs	Transformation method	No. of explants	No. of cas9 positive plants	Plants with active Cas9	Plants with small indels	Distinct small mutations/indels	Affected target site(s)	Small indels with frameshift	Plants with deletions between target sites	Distinct deletion(s) between target sites	Fragment deletions with frameshift
t1/tA	Biolistic	255	0	0	0	0	–	0	0	0	0
t1/tB	Biolistic	240	0	0	0	0	–	0	0	0	0
t2/tA	Biolistic	210	0	0	0	0	–	0	0	0	0
t2/tB	Biolistic	240	0	0	0	0	–	0	0	0	0
t3/tA	Biolistic	315	4	3	3	7	t3	4	0	0	0
t3/tB	Biolistic	270	4	4	4	6	t3	4	0	0	0
t1/tA	*Agrobacterium*-mediated	210	3	2	2	8	t1, tA	6	0	0	0
t1/tB	*Agrobacterium*-mediated	165	0	0	0	0	–	0	0	0	0
t2/tA	*Agrobacterium*-mediated	140	4	4	4	7^∗^	t2	5	0	0	0
t2/tB	*Agrobacterium*-mediated	90	4	1	1	1	tB	0	0	0	0
t3/tA	*Agrobacterium*-mediated	330	9	8	6	6	t3, tA	6	4	3	2
t3/tB	*Agrobacterium*-mediated	75	3	3	2	3	t3	2	2	3	2


*^∗^One indel is 87 bp long, but affects only one target site (t2).*

### Analysis of Site-specific Mutations and Fragment Deletions

**FIGURE 3:**
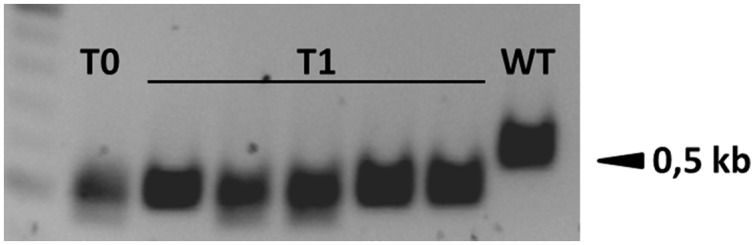
**Fragment deletions between two target sites were easily detected by PCR because the size of the amplicon was reduced by 90–139 bp in plant B411 and its T1 progeny.** WT, wild-type.

**FIGURE 4:**
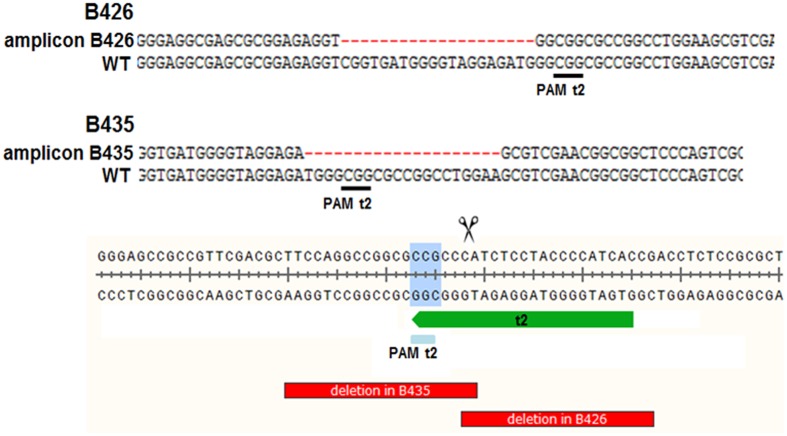
**Two T0 plants (B426 and B435) carrying 20 nt deletions.** In both cases the DSB occurred 3–4 nt upstream of the PAM, but the deletion was orientated in opposite directions. In plant B435, the NGG-PAM sequence was also lost. WT, wild-type reference DNA nucleotide sequence.

The analysis of plants transformed with pairs of sgRNAs revealed that indels were created with all sgRNA combinations, except t1/tB. However, DSBs were not always induced at both target sites, and one site often remained intact (**Table 2**). Fragment deletions induced by combinations of distinct sgRNAs were only observed in the transformants generated using *A. tumefaciens* for combinations t3/tA and t3/tB. These events were easily detected by PCR because the size of the amplicon was reduced by 90–139 bp (**Figure 3**). Six plants (B411, B413, B420, B423, B433, B439) were identified with deletions of DNA fragments between distinct target sites (**Table 2** and Supplementary Data Sheet S1). In two of these plants (B411 and B439) more than one fragment deletion was found within a single T0 individual (Supplementary Table S1). The DSBs occurred either specifically 3–4 nt upstream of the PAM (**Figure 2B1**), or at imprecise sites thus eliminating the genomic sequence beyond the PAMs (**Figure 2B2**). Interestingly, we detected a deletion of 20 bp in plants B426 and B435, which were obtained from the same experiment (combination t2/tA). In both cases the DSB occurred 3–4 nt upstream of the PAM, but the deletion was orientated differently in each plant, in one case (B426) extending downstream of the DSB and in the other case (B435) extending upstream (**Figure 4**). In the latter case, the NGG-PAM sequence was also lost.

### Monoallelic and Biallelic Mutations and Chimeric Lines

**FIGURE 5:**
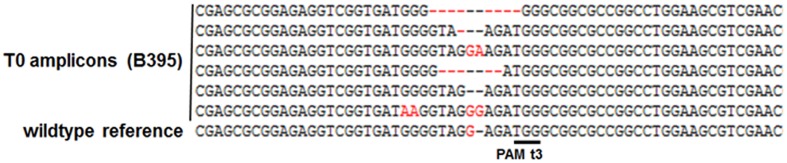
**Primary transgenic plants are often chimeric: six different mutation patterns were detected in T0 plant B395**.

We analyzed the site-specific mutations by aligning the sequences of 4–18 PCR amplicons (produced using primers ENG-F and ENG-R) per primary transformant against the reference wild-type barley DNA sequence. The 25 primary transformants contained 45 distinct site-specific mutations, 30 of which were frameshift mutations and most involved deletions (Supplementary Table S1). However, due to the limited number of amplicons analyzed per transformant the actual number of mutations might be higher. In seven of the plants, only the wild-type ENGase sequence was detected, suggesting that cas9 was inactive in these events. In 12 of the primary transformants, three or more mutation patterns were identified by genomic analysis, suggesting the plants were chimeric (**Figure 5** and Supplementary Table S1). Nine plants were identified with three or more types of mutation at a single target site: B351, B373, B374, B395, B396, B420, B427, B429, and B438 (Supplementary Data Sheet S1). In eight of the plants (B395, B411, B413, B420, B421, B423, B436, and B439) there was no evidence of the wild-type DNA sequence in at least six amplicons, suggesting these lines consisted only of transgenic, ENGase edited tissue.

Interestingly, a fragment deletion between two target sites was detected in plant B413, which was generated using sgRNAs t3/tA delivered by *A. tumefaciens* (**Table 2** and Supplementary Data Sheet S1). DSBs were induced simultaneously at sites t3 and tA (3–4 nt upstream of the corresponding PAMs) and the intervening stretch of DNA was eliminated (with no frameshift) apparently at both alleles, since no wild-type sequence was detected. Junction PCR was carried out to determine whether the T-DNA containing the cas9 gene was integrated at the DSB (data not shown), which would destroy one of the alleles, making the detection of mutations impossible. The target area was also amplified using primers that anneal further away. These experiments indicated the absence of integrated T-DNA or the presence of a large deletion. Furthermore, the cas9 gene segregated in the T1 progeny, suggesting that no allele was modified in such way that it could no longer be detected by PCR. Biallelic fragment deletions between selected target sites were also identified in plant B411 (Supplementary Table S1 and Data Sheet S1) created using the same combination of sgRNAs t3 and tA. However, although the DSBs in one allele were induced 3–4 nt upstream of the respective PAM, in the other the deletion was more extended and both NGG sequences were deleted (**Figure 2**). Multiallelic and probably chimeric fragment deletions were detected in plant B439 (Supplementary Table S1 and Data Sheet S1) created using the sgRNA combination t3 and tB delivered by *A. tumefaciens*. Here the elimination of two (of three) DNA sequences from between the target sites resulted in frameshifts. The initiation of the DSB at the t3 site was in all three cases atypical for Cas9.

Interestingly, in two T0 plants from variants t3/tA (B410) and t1/tA (B440) with small indels at both target sites, the mutation at the second target site reconstituted the open reading frame, possibly leaving the ENGase active. Fragment deletions between selected target sites also led to frameshifts, thus generating gene knock out candidates in four events (B411, B413, B420, B423, B433, and B439). In total, six variants of fragment deletions between target sites (with or without frameshift mutations) were detected.

When working with embryogenic tissue cultures, it is not always certain that only one plant is taken from a single callus because the tissue material often disintegrates and is distributed on the solid medium. In addition, Cas9 is considered active during callus development thus generating new mutations. Following a thorough analysis of the mutation types, clones derived from the same primordial transgenic cells were identified among the variants. Some plants were potentially partial clones, e.g., plants B413, B420, and B423 all contain the same 90-nt deletion between sites t3 and tA, but contain in addition different unique small indels. The primary transformants B389 and B393 could be considered as identical clones, because they share the same 3-nt deletion and derive from the same transformation experiment.

### Inheritance and Consistency of Targeted Mutations in the T1 Generation

**FIGURE 6:**
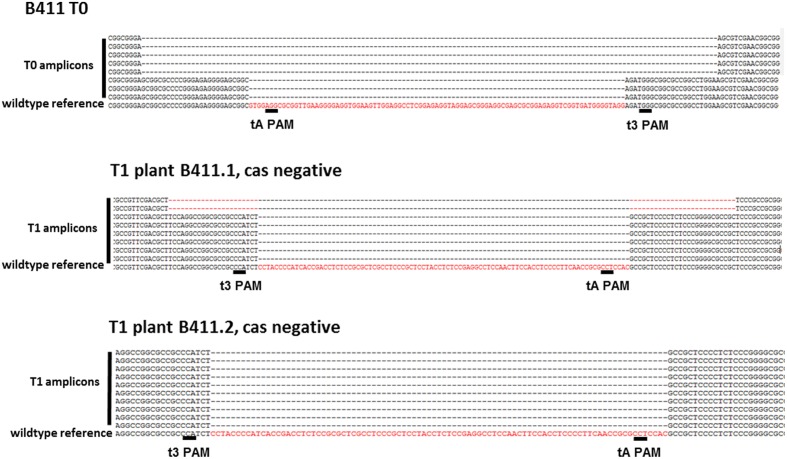
**T1 offspring from plant B411.** The T1 plants shown in the figure carry modifications on both alleles. B411.1 carries both of the frameshift deletions identified in the T0 parent. B411.2 carries one of the deletions at both alleles of the putative barley ENGase locus. Both types of deletion cause a frameshift. In both plants, the cas9:hpt transgene was absent due to independent segregation.

T1 progeny plants were obtained by self-fertilizing the primary transformants B395, B396, B411, B413, and B420 to determine whether the Cas9-induced mutations were heritable. We found that both indels and small fragment deletions between two target sites were inherited by the T1 progeny in all selected candidate lines (Supplementary Data Sheet S1), and the transgene (cas9:hpt) segregated independently from the mutations (Supplementary Figure S1). Both in-frame and knock out mutations were identified in the T1 generation. Transgene-free (cas9:hpt negative), biallelic ENGase edited lines were selected in T1 plants derived from the primary transformants B395, B411 B413, and B420. T1 plants either produced identical amplicons carrying a single type of mutation (B395.1) or distinct frameshift deletions at both alleles (e.g., B411.1; **Figure 6**). In the progeny of primary transformant B413, one homozygous transgene-free plant was identified that carried a deletion between the target sites that had already been identified in the T0 parent (B413.2). In other offspring from the same plant, a novel mutation was detected in the T1 generation, suggesting that the primary transformant was chimeric and/or an additional mutation had occurred later (B413.1, Supplementary Data Sheet S1). None of the transgenic barley plants showed macroscopic changes in phenotype compared to wild-type plants grown under the same conditions over two generations.

## Discussion

We designed five different sgRNAs for the Cas9-mediated knock out of the putative barley ENGase gene. In each genetic transformation experiment, at least two 20-nt sites were targeted, separated by ∼90 nt of intervening DNA. The site-specific mutations were detected by PCR and it should be noted that they were characterized by sequencing without the need for mutation enrichment using restriction enzymes or the T7El assay. Our data suggest that the CRISPR/Cas9 system generated DSBs and subsequent mutations in the stably transformed barley plants with an efficiency of at least 78%. A previous study in barley using *Agrobacterium*-mediated transfer of the cas9-construct reported a frequency of 10–23% of Cas9-induced mutations in the first generation (Lawrenson et al., 2015). From the total of 45 different Cas9-induced site-specific mutations detected among all variants, 66.7% proved to be frameshift mutations with a potential to generate homozygous knock out lines for the putative barley ENGase gene. Small deletions were the most frequent type of mutation we observed, consistent with the error-prone repair of DSBs by NHEJ (Manova and Gruszka, 2015; Pacher and Puchta, 2016). In agreement with previous studies, we confirmed that cleavage efficiency was highly dependent on the selected target site, and cleavage efficiency differed considerably even between adjacent sites (Wang T. et al., 2014; Johnson et al., 2015). In wheat suspension cell cultures, 11–12% of mutations were reported at two selected target sites and the complete deletion of the intervening region was observed in 2.8% of the events (Upadhyay et al., 2013). A high GC content can also affect the mutation efficiency. Zhang et al. (2014) tested 11 sgRNAs, and those with a higher GC content showed a higher editing efficiency. We also found that certain target sites were favored (t3 and tA) and the most efficient sgRNA proved to be t3 on the antisense DNA strand. The small indel mutation frequencies varied between the target sites, with t3 having the most (48.9%), tB the least (2.2%) and the other valued in between: t1 (6.7%), t2 (15.6%), tA (13.3%). When applying sgRNA combinations t3/tA and t3/tB, the deletion of the intervening fragment was observed in 6.7% of events. We did not investigate off-target mutations at sites with highly similar sequences. However, the frequency of off-target mutations induced by genome editing nucleases is typically well below that caused by chemical and physical mutagenesis (Podevin et al., 2013). Furthermore, any off-target modifications can be removed by segregation, if necessary.

Error-prone NHEJ can achieve precise repairs but typically introduces indels of 1–4 bp due to the annealing of single strands with short regions of microhomology (Lieber, 2010). This process is not the same as microhomology-mediated end joining (MMEJ), in which homologous sequences 5–25 bp in length are used to repair open DNA strands, frequently producing longer deletions at the DSB site (McVey and Lee, 2008). The mutations in our plants were typically short deletions typical of NHEJ, but we also observed longer deletions of up to 87 bp at single target sites reminiscent of MMEJ. We found that deletions were much more common than insertions. The high degree of chimerism we observed occasionally suggests that multiple targeted gene modification events occurred relatively late in callus development, and/or that multiple cells with different genetic transformation events were incorporated during the production of a single transformant. This is consistent with the findings reported by Feng et al. (2014). In contrast, transgenic plants with one type of mutation or biallelic mutations resulting from a single genetic transformation and mutation event probably derive from single embryogenic barley cells.

The deletion of ∼100-bp DNA segments between two target sites occurred even when transformation was achieved using a mixture of two *A. tumefaciens* cultures carrying the respective cas9 and sgRNA constructs. The elimination of a DNA segment requires the simultaneous formation of two DSBs at different target sites followed by NHEJ to join the distal free ends, excising the intermediate fragment. Interestingly, the Cas9 protein guided by the sgRNA did not always cleave the target DNA strand 3–4 nt upstream of the PAM (Supplementary Table S1). In approximately half of the events, the editing pattern was atypical. However, many DSBs probably occur as expected, but depending on the type of repair mechanism the deletion might be extended by exonucleases, resulting in an irregular cleavage pattern. It is generally accepted that the choice of a repair pathway and its action is also dependent on the type of the cell, its proliferation status and its cell cycle stage (Manova and Gruszka, 2015), thus the timing of the mutation may account for some of the differences. In four primary transformants from variant t3/tA (B411, B413, B420, B423), typical DSBs were introduced 3–4 nt upstream of the PAM, followed by the elimination of the intervening DNA sequence. Plants B413, B420, and B423 lost the same 90 nt fragment and are likely to be partial clones. However, they contained additional unique indels. Furthermore, plant B411 was characterized by a second 139-nt imprecise deletion which was trimmed in a manner that was atypical for Cas9. Interestingly, three different imprecise fragment deletions between sites t3 and tB were detected in plant B439, where the DSBs and the excised DNA segments were irregular. In these events, the protospacer sequence was destroyed after Cas9-induced cleavage and no successive genome editing would be possible. However, the three types of DNA fragment eliminations seem to feature the same trigger and pattern.

One advantage of segment deletions between a pair of target sites is that they can easily be detected by PCR. This editing method could therefore be used for screening in transient expression systems. There is a growing demand for the DNA-free delivery of CRISPR system components directly as functional sgRNA and Cas9 protein, in order to avoid the genomic integration of cas9/sgRNA transgenes and the need for segregation, but this would also mean that selectable markers cannot be used for the initial identification of candidate mutants. In such case, the elimination of a DNA fragment from the selected target area would facilitate the selection of edited lines directly by diagnostic genomic PCR. This would, however, require the careful design and selection of sgRNAs, which must trigger the editing of target sites with reasonable efficiency.

Several groups have already reported the induction of biallelic or homozygous mutations directly in the primary transformants using the CRISPR/Cas9 system confirming the high efficiency of this genome editing platform (Shan et al., 2013; Brooks et al., 2014; Zhang et al., 2014; Zhou et al., 2014; Zhu et al., 2017). Indeed, we also observed putative biallelic modifications in the T0 generation, which were verified by genotyping in the corresponding T1 lines. For example, biallelic frameshift mutations were confirmed in the T1 progeny derived from plant B411. The ENGase mutations had segregated from the cas9 transgene in these T1 plants, confirming that the cas9:sgRNA T-DNA had integrated on a separate chromosome, and we could still detect both frameshift mutations in the T1 generation. In contrast, plant B413 was initially regarded as potentially homozygous for a fragment deletion, but then a novel mutation was detected in some T1 individuals suggesting the primary transformant may have been chimeric (Supplementary Data Sheet S1). The definitive genotype of the primary transformants can therefore be confirmed only following the genetic analysis of T1 segregants. The proportion of homozygous transgene-free mutants in the offspring could be increased using embryogenic pollen cultures, which are well established in barley (Kapusi et al., 2013).

Knock out lines for selected genes are useful for the analysis of gene function, and the straightforward generation of such lines using CRISPR/Cas9 highlights the potential of this technology for functional genetics in both model plants and crops. Our transgenic barley plants with biallelic or homozygous frameshift mutations in the putative ENGase gene did not show any macroscopic differences in phenotype compared to wild-type plants as anticipated (Fischl et al., 2011). The resulting plants will be investigated at the biochemical level to investigate possible changes in the N-glycan composition of endogenous and recombinant proteins as well as any effects on endosperm physiology.

## Author Contributions

EK and ES designed the study. EK and MC-G carried out the experiments. SM produced and provided data. EK and ES wrote the manuscript.

## Conflict of Interest Statement

The authors declare that the research was conducted in the absence of any commercial or financial relationships that could be construed as a potential conflict of interest.
