# Supplementary material for: Heritable Genomic Fragment Deletions and Small Indels in the Putative ENGase Gene Induced by CRISPR/Cas9 in Barley
Source: Front Plant Sci. 2017 Apr 25;8:540. doi: 10.3389/fpls.2017.00540 (PMC5404177; doi:10.3389/fpls.2017.00540)
Supplement: Supplementary file 1 [file Table_1.DOCX]

Table S1. List of plants analyzed
