# Supplementary figures and images for: Heritable Genomic Fragment Deletions and Small Indels in the Putative ENGase Gene Induced by CRISPR/Cas9 in Barley
Source: Front Plant Sci. 2017 Apr 25;8:540. doi: 10.3389/fpls.2017.00540 (PMC5404177; doi:10.3389/fpls.2017.00540)

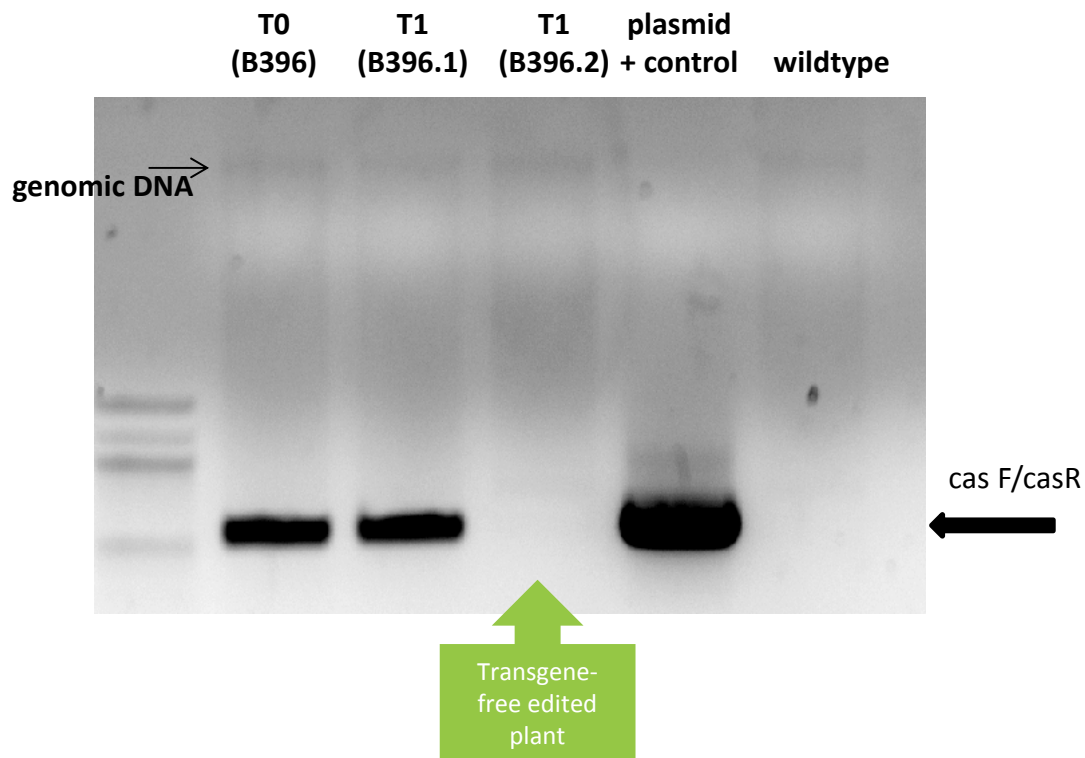

Figure S1. T1 plants were tested for the presence of the *cas9* gene using primers Cas-F and Cas-R (Table 1).

Supplement: Supplementary file 2 [file Image_1.pdf]
