# Supplementary material for: Heritable Genomic Fragment Deletions and Small Indels in the Putative ENGase Gene Induced by CRISPR/Cas9 in Barley
Source: Front Plant Sci. 2017 Apr 25;8:540. doi: 10.3389/fpls.2017.00540 (PMC5404177; doi:10.3389/fpls.2017.00540)

B351 T0 ENG1 contigs

T0 amplicons

```
ACGCTTCCAGGCCGG----------------CCCCATCACCGAC
ACGCTTCCAGGCCGGCGCCGCCCA--T-CTACCCCATCACCGAC
ACGCTTCCAGGCCGGCGCCGCCCATCTCCTACCCCATCACCGAC
ACGCTTCCAGGCCGGCGCCGCCCAT------CCCCATCACCGAC
ACGCTTCCAGGCCGGCGCCGCCCATCTCCTACCCCATCACCGAC
ACGCTTCCAGGCCGGCGCCGCCCATCTCCTACCCCATCACCGAC
ACGCTTCCAGGCCGGCGCCGCCCATCT-CTACCCCATCACCGAC
ACGCTTCCAGGCCGGCGCCGCCCATCTCCTACCCCATCACCGAC
ACGCTTCCAGGCCGGCGCCGCCCATCTCCTACCCCATCACCGAC
ACGCTTCCAGGCCGGCGCCGCCCATCTCCTACCCCATCACCGAC
```

wildtype reference

```
ACGCTTCCAGGCCGGCGCCGCCCATCTCCTACCCCATCACCGAC
```

t3 PAM

B373 T0 ENG1 contigs

T0 amplicons

```
GACGCTTCCAGGCCGGCGCCGCCCATCTCCTACCCCATCACCGACCTCTCC
GACGCTTCCAGGCCGGCGCCGCCCATCTCCTACCCCATCACCGACCTCTCC
GACGCTTCCAGGCCGGCGCCGCCCATCTCCTACCCCATCACCGACCTCTCC
GACGCTTCCAGGCCGGCGCCGCC---------------CACCGACCTCTCC
GACGCTTCCAGGCCGGCGCCGCCCATCTCCTACCCCATCACCGACCTCTCC
GACGCTTCCAGGCCGGCGCCGCCCATCTCCTACCCCATCACCGACCTCTCC
GACGCTTCCAGGCCGGCGCCGCCCATCTCCTACCCCATCACCGACCTCTCC
GACGCTTCCAGGCCGGCGCCGCCCATCTCCTACCCCATCACCGACCTCTCC
GACGCTTCCAGGCCGGCGCCGCCCATCT--TACCCCATCACCGACCTCTCC
GACGCTTCCAGGCCGGCGCCGCCCATCT-CTACCCCATCACCGACCTCTCC
GACGCTTCCAGGCCGGCGCCGCCCATCTCCTACCCCATCACCGACCTCTCC
GACGCTTCCAGGCCGGCGCCGCCCATCTCCTACCCCATCACCGACCTCTCC
GACGCTTCCAGGCCGGCGCCGCCCAT---CTACCCCATCACCGACCTCTCC
GACGCTTCCAGGCCGGCGCCGCCCATCTCCTACCCCATCACCGACCTCTCC
GACGCTTCCAGGCCGGCGCCGCCCATCTCCTACCCCATCACCGACCTCTCC
GACGCTTCCAGGCCGGCGCCGCC---------CCCCATCACCGACCTCTCC
GACGCTTCCAGGCCGGCGCCGCCCATCTCCTACCCCATCACCGACCTCTCC
GACGCTTCCAGGCCGGCGCCGCCCATCTCCTACCCCATCACCGACCTCTCC
```

wildtype reference

```
GACGCTTCCAGGCCGGCGCCGCCCATCTCCTACCCCATCACCGACCTCTCC
```

t3 PAM

B374 T0 ENG1 contigs

T0 amplicons

```
GCCGTTCGACGCTTCCAGGCCGGCGCCGCCCAT---CTACCCCATCACCGACC
GCCGTTCGACGCTTCCAGGCCGGCGCCGCCCATCT-CTACCCCATCACCGACC
GCCGTTCGACGCTTCCAGGCCGGCGCCGCCCATCTCCTACCCCATCACCGACC
GCCGTTCGACGCTTCCAGGCCGGCGCCGCCCATCTCCTACCCCATCACCGACC
GCCGTTCGACGCTTCCAGGCCGGCGCCGCCCATCTCCTACCCCATCACCGACC
GCCGTTCGACGCTTCCAGGCCGGCGCCGCCCATCTCCTACCCCATCACCGACC
GCCGTTCGACGCTTCCAGGCCGGCGCCGCCCATCTCCTACCCCATCACCGACC
GCCGTTCG------------------------------ACCCCATCACCGACC
GCCGTTCGACGCTTCCAGGCCGGCGCCGCCCATCTCCTACCCCATCACCGACC
GCCGTTCGACGCTTCCAGGCCGGCGCCGCCCATCTCCTACCCCATCACCGACC
GCCGTTCGACGCTTCCAGGCCGGCGCCGCCCATCTCCTACCCCATCACCGACC
GCCGTTCGACGCTTCCAGGCCGGCGCCGCCCATCTCCTACCCCATCACCGACC
GCCGTTCGACGCTTCCAGGCCGGCGCCGCCCATCTCCTACCCCATCACCGACC
```

wildtype reference

```
GCCGTTCGACGCTTCCAGGCCGGCGCCGCCCATCTCCTACCCCATCACCGACC
```

t3 PAM

B389 T0 ENG1 contigs

T0 amplicons

```
AGGTCGGTGATGGGGTAG---ATGGGCGGCGCCGGCCTGGAAG
AGGTCGGTGATGGGGTAG---ATGGGCGGCGCCGGCCTGGAAG
AGGTCGGTGATGGGGTAGGAGATGGGCGGCGCCGGCCTGGAAG
AGGTCGGTGATGGGGTAGGAGATGGGCGGCGCCGGCCTGGAAG
AGGTCGGTGATGGGGTAGGAGATGGGCGGCGCCGGCCTGGAAG
AGGTCGGTGATGGGGTAG---ATGGGCGGCGCCGGCCTGGAAG
```

wildtype reference

```
AGGTCGGTGATGGGGTAGGAGATGGGCGGCGCCGGCCTGGAAG
```

t3 PAM

B393 T0 ENG1 contigs

T0 amplicons

```
GAGGTCGGTGATGGGGTAG---ATGGGCGGCGCCGGCCT
GAGGTCGGTGATGGGGTAG---ATGGGCGGCGCCGGCCT
GAGGTCGGTGATGGGGTAGGAGATGGGCGGCGCCGGCCT
GAGGTCGGTGATGGGGTAG---ATGGGCGGCGCCGGCCT
GAGGTCGGTGATGGGGTAGGAGATGGGCGGCGCCGGCCT
```

wildtype reference

```
GAGGTCGGTGATGGGGTAGGAGATGGGCGGCGCCGGCCT
```

t3 PAM

B395 T0 ENG1 contigs

T0 amplicons

```
CGGTGATGGGGTA---AGATGGGCGGCGC
CGGTGATGGG----------GGGCGGCGC
CGGTGATGGGGTA---AGATGGGCGGCGC
CGGTGATGGGGTAGGAAGATGGGCGGCGC
CGGTGATGGGG-------ATGGGCGGCGC
CGGTGATGGGGTAG--AGATGGGCGGCGC
CGGTGATAAGGTAGGGAGATGGGCGGCGC
```

wildtype reference

```
CGGTGATGGGGTAGG-AGATGGGCGGCGC
```

t3 PAM

T1 ENG1 contigs B395.1 (cas9 negative)

T1 amplicons

```
CGGTGATGGGGTAG-AGATGGGCGGCGC
CGGTGATGGGGTAG-AGATGGGCGGCGC
CGGTGATGGGGTAG-AGATGGGCGGCGC
CGGTGATGGGGTAG-AGATGGGCGGCGC
CGGTGATGGGGTAG-AGATGGGCGGCGC
CGGTGATGGGGTAG-AGATGGGCGGCGC
```

wildtype reference

```
CGGTGATGGGGTAGGAGATGGGCGGCGC
```

t3 PAM

B396 T0 ENG1 contigs

```
T0 amplicons
CGGTGATGGGGTAGGAGATGGGCGGCGC
CGGTGATGGGGTAG---ATGGGCGGCGC
CGGTGATGGGGTAG---ATGGGCGGCGC
CGGTGATGGGGTAGGAGATGGGCGGCGC
CGGTGATGGGGTAG---ATGGGCGGCGC
CGGTGATGGGGTAGGAGATGGGCGGCGC
wildtype reference
CGGTGATGGGGTAGGAGATGGGCGGCGC
```

t3 PAM

T1 ENG1 contigs B396.1 (cas9 positive)

```
T1 amplicons
CGGTGATGGGGTAG---ATGGGCGGCGC
CGGTGATGGGGTAG---ATGGGCGGCGC
CGGTGATGGGGTAG---ATGGGCGGCGC
CGGTGATGGGGTAG---ATGGGCGGCGC
CGGTGATGGGGTAG---ATGGGCGGCGC
CGGTGATGGGGTAG---ATGGGCGGCGC
wildtype reference
CGGTGATGGGGTAGGAGATGGGCGGCGC
```

t3 PAM

T1 ENG1 contigs B396.2 (cas9 negative)

```
T1 amplicons
CGGTGATGGGGTAGGAGATGGGCGGCGC
CGGTGATGGGGTAG---ATGGGCGGCGC
CGGTGATGGGGTAG---ATGGGCGGCGC
CGGTGATGGGGTAGGAGATGGGCGGCGC
CGGTGATGGGGTAG---ATGGGCGGCGC
CGGTGATGGGGTAG---ATGGGCGGCGC
wildtype reference
CGGTGATGGGGTAGGAGATGGGCGGCGC
```

t3 PAM

B410 T0 ENG1 contigs

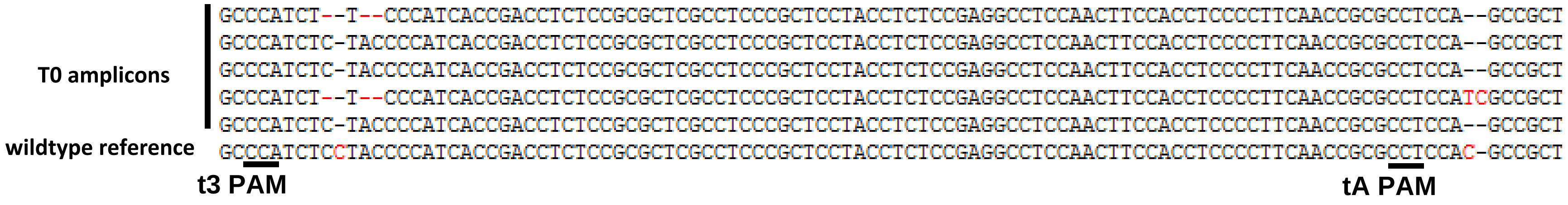

B411 T0 ENG1 contigs

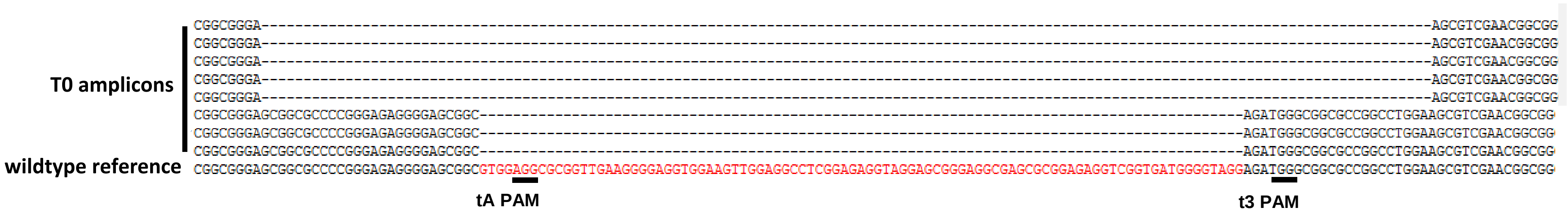

T1 ENG1 contigs B411.1 (cas9 negative)

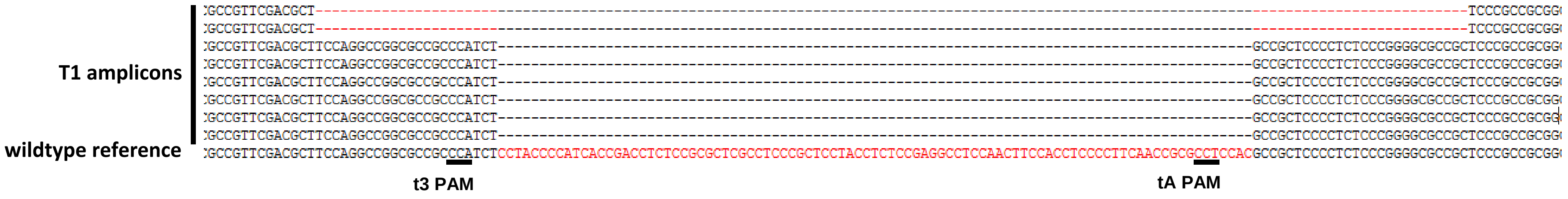

T1 ENG1 contigs B411.2 (cas9 negative)

T1 amplicons

```
AGGCCGGCGCCGCCCATCT-------------------------------------------------------------------------------------------GCCGCTCCCCTCTCCCGGGGCGC
AGGCCGGCGCCGCCCATCT-------------------------------------------------------------------------------------------GCCGCTCCCCTCTCCCGGGGCGC
AGGCCGGCGCCGCCCATCT-------------------------------------------------------------------------------------------GCCGCTCCCCTCTCCCGGGGCGC
AGGCCGGCGCCGCCCATCT-------------------------------------------------------------------------------------------GCCGCTCCCCTCTCCCGGGGCGC
AGGCCGGCGCCGCCCATCT-------------------------------------------------------------------------------------------GCCGCTCCCCTCTCCCGGGGCGC
AGGCCGGCGCCGCCCATCT-------------------------------------------------------------------------------------------GCCGCTCCCCTCTCCCGGGGCGC
AGGCCGGCGCCGCCCATCT-------------------------------------------------------------------------------------------GCCGCTCCCCTCTCCCGGGGCGC
AGGCCGGCGCCGCCCATCT-------------------------------------------------------------------------------------------GCCGCTCCCCTCTCCCGGGGCGC
AGGCCGGCGCCGCCCATCT-------------------------------------------------------------------------------------------GCCGCTCCCCTCTCCCGGGGCGC
AGGCCGGCGCCGCCCATCT-------------------------------------------------------------------------------------------GCCGCTCCCCTCTCCCGGGGCGC
```

wildtype reference

```
AGGCCGGCGCCGCCCATCTCCTACCCCATCACCGACCTCTCCGCGCTCGCCTCCCGCTCCTACCTCTCCGAGGCCTCCAACTTCCACCTCCCCTTCAACCGCGCCTCCACGCCGCTCCCCTCTCCCGGGGCGC
```

t3 PAM　　tA PAM

B413 T0 ENG1 contigs

T0 amplicons

```
AGGGGAGCGGCG------------------------------------------------------------------------------------------AGATGGGCGG
AGGGGAGCGGCG------------------------------------------------------------------------------------------AGATGGGCGG
AGGGGAGCGGCG------------------------------------------------------------------------------------------AGATGGGCGG
AGGGGAGCGGCG------------------------------------------------------------------------------------------AGATGGGCGG
AGGGGAGCGGCG------------------------------------------------------------------------------------------AGATGGGCGG
AGGGGAGCGGCG------------------------------------------------------------------------------------------AGATGGGCGG
AGGGGAGCGGCG------------------------------------------------------------------------------------------AGATGGGCGG
AGGGGAGCGGCG------------------------------------------------------------------------------------------AGATGGGCGG
AGGGGAGCGGCG------------------------------------------------------------------------------------------AGATGGGCGG
AGGGGAGCGGCG------------------------------------------------------------------------------------------AGATGGGCGG
```

wildtype reference

```
AGGGGAGCGGCGTGGAGGCGCGGTTGAAGGGGAGGTGGAAGTTGGAGGCCTCGGAGAGGTAGGAGCGGGAGGCGAGCGCGGAGAGGTCGGTGATGGGGTAGGAGATGGGCGG
```

tA PAM　　t3 PAM

T1 ENG1 contigs B413.1

T1 amplicons

```
GGAGAGGGGAGC----TGGAGGCGCGGTTGAAGGGGAGGTGGAAGTTGGAGGCCTCGGAGAGGTAGGAGCGGGAGGCGAGCGCGGAGAGGTCGGTGATGGGGTA--AGATGGGCGGCGCCGG
GGAGAGGGGAGC----TGGAGGCGCGGTTGAAGGGGAGGTGGAAGTTGGAGGCCTCGGAGAGGTAGGAGCGGGAGGCGAGCGCGGAGAGGTCGGTGATGGGGTA--AGATGGGCGGCGCCGG
GGAGAGGGGAGC----TGGAGGCGCGGTTGAAGGGGAGGTGGAAGTTGGAGGCCTCGGAGAGGTAGGAGCGGGAGGCGAGCGCGGAGAGGTCGGTGATGGGGTA--AGATGGGCGGCGCCGG
GGAGAGGGGAGC----TGGAGGCGCGGTTGAAGGGGAGGTGGAAGTTGGAGGCCTCGGAGAGGTAGGAGCGGGAGGCGAGCGCGGAGAGGTCGGTGATGGGGTA--AGATGGGCGGCGCCGG
GGAGAGGGGAGC----TGGAGGCGCGGTTGAAGGGGAGGTGGAAGTTGGAGGCCTCGGAGAGGTAGGAGCGGGAGGCGAGCGCGGAGAGGTCGGTGATGGGGTA--AGATGGGCGGCGCCGG
GGAGAGGGGAGC----TGGAGGCGCGGTTGAAGGGGAGGTGGAAGTTGGAGGCCTCGGAGAGGTAGGAGCGGGAGGCGAGCGCGGAGAGGTCGGTGATGGGGTA--AGATGGGCGGCGCCGG
```

wildtype reference

```
GGAGAGGGGAGCGGCGTGGAGGCGCGGTTGAAGGGGAGGTGGAAGTTGGAGGCCTCGGAGAGGTAGGAGCGGGAGGCGAGCGCGGAGAGGTCGGTGATGGGGTAGGAGATGGGCGGCGCCGG
```

tA PAM　　t3 PAM

T1 ENG1 contigs B413.2 (cas9 negative)

```
T1 amplicons        AGAGGGGAGCGGCG------------------------------------------------------------------------------------------AGATGGGCGGCGCCGGC
                    AGAGGGGAGCGGCG------------------------------------------------------------------------------------------AGATGGGCGGCGCCGGC
                    AGAGGGGAGCGGCG------------------------------------------------------------------------------------------AGATGGGCGGCGCCGGC
                    AGAGGGGAGCGGCG------------------------------------------------------------------------------------------AGATGGGCGGCGCCGGC
                    AGAGGGGAGCGGCG------------------------------------------------------------------------------------------AGATGGGCGGCGCCGGC
                    AGAGGGGAGCGGCG------------------------------------------------------------------------------------------AGATGGGCGGCGCCGGC
                    AGAGGGGAGCGGCG------------------------------------------------------------------------------------------AGATGGGCGGCGCCGGC
                    AGAGGGGAGCGGCG------------------------------------------------------------------------------------------AGATGGGCGGCGCCGGC
                    AGAGGGGAGCGGCG------------------------------------------------------------------------------------------AGATGGGCGGCGCCGGC
wildtype reference  AGAGGGGAGCGGCGTGGAGGCGCGGTTGAAGGGGAGGTGGAAGTTGGAGGCCTCGGAGAGGTAGGAGCGGGAGGCGAGCGCGGAGAGGTCGGTGATGGGGTAGGAGATGGGCGGCGCCGGC
                           tA PAM                                                                               t3 PAM
```

B420 T0 ENG1 contigs

```
T0 amplicon         GGCGCCGCCCATCT-----------------------------------------------------------------------------------------CGCCGCTCCCCTCTC
wildtype reference  GGCGCCGCCCATCTCCTACCCATCACCGACCTCTCCGCGCTCGCCTCCCGCTCCTACCTCTCCGAGGCCTCCAACTTCCACCTCCCCTTCAACCGCGCCTCCACGCCGCTCCCCTCTC
                      t3 PAM                                                                                   tA PAM
```

```
T0 amplicons        GCCGCCCATCTT----CCCATCACCGACCTCTCCGCGCTCGCCTCCCGCTCCTACCTCTCCGAGGCCTCCAACTTCCACCTCCCCTTCAACCGCGCCTCCA-GCCGCTCC
                    GCCGCCCATCTT----CCCATCACCGACCTCTCCGCGCTCGCCTCCCGCTCCTACCTCTCCGAGGCCTCCAACTTCCACCTCCCCTTCAACCGCGCCTCCA-GCCGCTCC
                    GCCGCCCATCTT----CCCATCACCGACCTCTCCGCGCTCGCCTCCCGCTCCTACCTCTCCGAGGCCTCCAACTTCCACCTCCCCTTCAACCGCGCCTCCA-GCCGCTCC
                    GCCGCCCATCTT----CCCATCACCGACCTCTCCGCGCTCGCCTCCCGCTCCTACCTCTCCGAGGCCTCCAACTTCCACCTCCCCTTCAACCGCGCCTCCA-GCCGCTCC
wildtype reference  GCCGCCCATCTCCTACCCCATCACCGACCTCTCCGCGCTCGCCTCCCGCTCCTACCTCTCCGAGGCCTCCAACTTCCACCTCCCCTTCAACCGCGCCTCCACGCCGCTCC
                      t3 PAM                                                                                     tA PAM
```

```
T0 amplicon         GAGCGGC-TGGAGGCGCGGTTGAAGGGGAGGTGGAAGTTGGAGGCCTCGGAGAGGTAGGAGCGGGAGGCGAGCGCGGAGAGGTCGGTGATGGGGTAG-----GGGCGGCG
wildtype reference  GAGCGGCGTGGAGGCGCGGTTGAAGGGGAGGTGGAAGTTGGAGGCCTCGGAGAGGTAGGAGCGGGAGGCGAGCGCGGAGAGGTCGGTGATGGGGTAGGAGATGGGCGGCG
                         tA PAM                                                                                t3 PAM
```

T1 ENG1 contigs B420.1

```
T1 amplicons        GAGCGGCGGTGGAGGCGCGGTTGAAGGGGAGGTGGAAGTTGGAGGCCTCGGAGAGGTAGGAGCGGGAGGCGAGCGCGGAGAGGTCGGTGATGGGA----AGATGGGCGGC
                    GAGCGGCGGTGGAGGCGCGGTTGAAGGGGAGGTGGAAGTTGGAGGCCTCGGAGAGGTAGGAGCGGGAGGCGAGCGCGGAGAGGTCGGTGATGGGA----AGATGGGCGGC
                    GAGCGGCGGTGGAGGCGCGGTTGAAGGGGAGGTGGAAGTTGGAGGCCTCGGAGAGGTAGGAGCGGGAGGCGAGCGCGGAGAGGTCGGTGATGGGA----AGATGGGCGGC
                    GAGCGGCGGTGGAGGCGCGGTTGAAGGGGAGGTGGAAGTTGGAGGCCTCGGAGAGGTAGGAGCGGGAGGCGAGCGCGGAGAGGTCGGTGATGGGA----AGATGGGCGGC
                    GAGCGGCGGTGGAGGCGCGGTTGAAGGGGAGGTGGAAGTTGGAGGCCTCGGAGAGGTAGGAGCGGGAGGCGAGCGCGGAGAGGTCGGTGATGGGA----AGATGGGCGGC
                    GAGCGGCGGTGGAGGCGCGGTTGAAGGGGAGGTGGAAGTTGGAGGCCTCGGAGAGGTAGGAGCGGGAGGCGAGCGCGGAGAGGTCGGTGATGGGA----AGATGGGCGGC
wildtype reference  GAGCGGCG-TGGAGGCGCGGTTGAAGGGGAGGTGGAAGTTGGAGGCCTCGGAGAGGTAGGAGCGGGAGGCGAGCGCGGAGAGGTCGGTGATGGGGTAGGAGATGGGCGGC
                          tA PAM                                                                                 t3 PAM
```

T1 ENG1 contigs B420.2 (cas9 negative)

```
GGAGCGGC--TGGAGGCGCGGTTGAAGGGGAGGTGGAAGTTGGAGGCCTCGGAGAGGTAGGAGCGGGAGGCGAGCGCGGAGAGGTCGGTGATGGGGTAGGAGATGGGCGGCG
GGAGCGGC--TGGAGGCGCGGTTGAAGGGGAGGTGGAAGTTGGAGGCCTCGGAGAGGTAGGAGCGGGAGGCGAGCGCGGAGAGGTCGGTGATGGGGTAGGAGATGGGCGGCG
GGAGCGGC--TGGAGGCGCGGTTGAAGGGGAGGTGGAAGTTGGAGGCCTCGGAGAGGTAGGAGCGGGAGGCGAGCGCGGAGAGGTCGGTGATGGGGTAGGAGATGGGCGGCG
GGAGCGGC--TGGAGGCGCGGTTGAAGGGGAGGTGGAAGTTGGAGGCCTCGGAGAGGTAGGAGCGGGAGGCGAGCGCGGAGAGGTCGGTGATGGGGTAGGAGATGGGCGGCG
GGAGCGGCGGTGGAGGCGCGGTTGAAGGGGAGGTGGAAGTTGGAGGCCTCGGAGAGGTAGGAGCGGGAGGCGAGCGCGGAGAGGTCGGTGATGGGA----AGATGGGCGGCG
GGAGCGGC--TGGAGGCGCGGTTGAAGGGGAGGTGGAAGTTGGAGGCCTCGGAGAGGTAGGAGCGGGAGGCGAGCGCGGAGAGGTCGGTGATGGGGTAGGAGATGGGCGGCG
GGAGCGGCGGTGGAGGCGCGGTTGAAGGGGAGGTGGAAGTTGGAGGCCTCGGAGAGGTAGGAGCGGGAGGCGAGCGCGGAGAGGTCGGTGATGGGA----AGATGGGCGGCG
GGAGCGGCGGTGGAGGCGCGGTTGAAGGGGAGGTGGAAGTTGGAGGCCTCGGAGAGGTAGGAGCGGGAGGCGAGCGCGGAGAGGTCGGTGATGGGA----AGATGGGCGGCG
GGAGCGGCGGTGGAGGCGCGGTTGAAGGGGAGGTGGAAGTTGGAGGCCTCGGAGAGGTAGGAGCGGGAGGCGAGCGCGGAGAGGTCGGTGATGGGA----AGATGGGCGGCG
GGAGCGGCG-TGGAGGCGCGGTTGAAGGGGAGGTGGAAGTTGGAGGCCTCGGAGAGGTAGGAGCGGGAGGCGAGCGCGGAGAGGTCGGTGATGGGGTAGGAGATGGGCGGCG
```

tA PAM　　　t3 PAM

B421 T0 ENG1 contigs

```
                    CCCATCTT----CCCATCACCGACCTCTCCGCGCTCGCCTCCCGCTCCTACCTCTCCGAGGCCTCCAACTTCCACCTCCCCTTCAACCGCGCCTCCATCGCCGCTC
                    CCCATCTT----CCCATCACCGACCTCTCCGCGCTCGCCTCCCGCTCCTACCTCTCCGAGGCCTCCAACTTCCACCTCCCCTTCAACCGCGCCTCCATCGCCGCTC
                    CCCATCTT----CCCATCACCGACCTCTCCGCGCTCGCCTCCCGCTCCTACCTCTCCGAGGCCTCCAACTTCCACCTCCCCTTCAACCGCGCCTCCA-CGCCGCTC
T0 amplicons        CCCATCTT----CCCATCACCGACCTCTCCGCGCTCGCCTCCCGCTCCTACCTCTCCGAGGCCTCCAACTTCCACCTCCCCTTCAACCGCGCCTCCATCGCCGCTC
                    CCCATCTT----CCCATCACCGACCTCTCCGCGCTCGCCTCCCGCTCCTACCTCTCCGAGGCCTCCAACTTCCACCTCCCCTTCAACCGCGCCTCCATCGCCGCTC
                    CCCATCTT----CCCATCACCGACCTCTCCGCGCTCGCCTCCCGCTCCTACCTCTCCGAGGCCTCCAACTTCCACCTCCCCTTCAACCGCGCCTCCATCGCCGCTC
                    CCCATCTT----CCCATCACCGACCTCTCCGCGCTCGCCTCCCGCTCCTACCTCTCCGAGGCCTCCAACTTCCACCTCCCCTTCAACCGCGCCTCCA-CGCCGCTC
wildtype reference  CCCATCTCCTACCCCATCACCGACCTCTCCGCGCTCGCCTCCCGCTCCTACCTCTCCGAGGCCTCCAACTTCCACCTCCCCTTCAACCGCGCCTCCA-CGCCGCTC
```

t3 PAM　　　tA PAM

B422 T0 ENG1 contigs

T0 amplicons
```
GGAGAGGGGAGCGGC--TGGAGGCGCGGTTGAA
GGAGAGGGGAGCGGC--TGGAGGCGCGGTTGAA
GGAGAGGGGAGCGGC--TGGAGGCGCGGTTGAA
GGAGAGGGGAGCGGCG-TGGAGGCGCGGTTGAA
GGAGAGGGGAGCGGCG-TGGAGGCGCGGTTGAA
GGAGAGGGGAGCGGCGTTGGAGGCGCGGTTGAA
GGAGAGGGGAGCGGCG-TGGAGGCGCGGTTGAA
```
wildtype reference
```
GGAGAGGGGAGCGGCG-TGGAGGCGCGGTTGAA
```
tA PAM

B423 T0 ENG1 contigs

T0 amplicons
```
GGGAGCGGC-TGGAGGCGCGGTTGAAGGGGAGGTGGAAGTTGGAGGCCTCGGAGAGGTAGGAGCGGGAGGCGAGCGCGGAGAGGTCGGTGATGGGGTA-GAGATGGGCGGCGCCC
GGGAGCGGC------------------------------------------------------------------------------------------GAGATGGGCGGCGCCC
GGGAGCGGC-TGGAGGCGCGGTTGAAGGGGAGGTGGAAGTTGGAGGCCTCGGAGAGGTAGGAGCGGGAGGCGAGCGCGGAGAGGTCGGTGATGGGGTA-GAGATGGGCGGCGCCC
GGGAGCGGC------------------------------------------------------------------------------------------GAGATGGGCGGCGCCC
```
wildtype reference
```
GGGAGCGGCGTGGAGGCGCGGTTGAAGGGGAGGTGGAAGTTGGAGGCCTCGGAGAGGTAGGAGCGGGAGGCGAGCGCGGAGAGGTCGGTGATGGGGTAGGAGATGGGCGGCGCCC
```
tA PAM t3 PAM

B426 T0 ENG1 contigs

T0 amplicons
```
CGGAGAGGT--------------------GGCGGCGCCGG
CGGAGAGGTCGGTGATGGGGTAGGAGATGGGCGGCGCCGG
CGGAGAGGT--------------------GGCGGCGCCGG
CGGAGAGGTCGGTGATGGGGTAGGAGATGGGCGGCGCCGG
CGGAGAGGT--------------------GGCGGCGCCGG
CGGAGAGGT--------------------GGCGGCGCCGG
CGGAGAGGTCGGTGATGGGGTAGGAGATGGGCGGCGCCGG
```
wildtype reference
```
CGGAGAGGTCGGTGATGGGGTAGGAGATGGGCGGCGCCGG
```
t2 PAM

B427 T0 ENG1 contigs

T0 amplicons

```
GGGGTAGGAG-----GCGGCGCCGGCCTGGAAGCGTCGAACGGCGG
GGGGTAGGAGAT-GGGCGGCGCCGGCCTGGAAGCGTCGAACGGCGG
GGGGTAGGAGATTGGGCGGCGCCGGCCTGGAAGCGTCGAACGGCGG
GGGGTAGGAGAT-GGGCGGCGCCGGCCTGGAAGCGTCGAACGGCGG
GGGGTAGGAGAT-GGGCGGCGCCGGCCTGGAAGCGTCGAACGGCGG
GGGGTAGGAGA----------------------------ACGGCGG
GGGGTAGGAGAT-GGGCGGCGCCGGCCTGGAAGCGTCGAACGGCGG
GGGGTAGGAGAT-GGGCGGCGCCGGCCTGGAAGCGTCGAACGGCGG
GGGGTAGGAGAT-GGGCGGCGCCGGCCTGGAAGCGTCGAACGGCGG
```

wildtype reference

```
GGGGTAGGAGAT-GGGCGGCGCCGGCCTGGAAGCGTCGAACGGCGG
```

t2 PAM

B428 T0 ENG1 contigs

T0 amplicons

```
GGTGATGGGGTAG-AGATGGGCGGCGC
GGTGATGGGGTAG-AGATGGGCGGCGC
GGTGATGGGGTAGGAGATGGGCGGCGC
GGTGATGGGGTAGGAGATGGGCGGCGC
GGTGATGGGGTAGGAGATGGGCGGCGC
GGTGATGGGGTAGGAGATGGGCGGCGC
GGTGATGGGGTAGGAGATGGGCGGCGC
GGTGATGGGGT----GATGGGCGGCGC
```

wildtype reference

```
GGTGATGGGGTAGGAGATGGGCGGCGC
```

t3 PAM

B429 T0 ENG1 contigs

T0 amplicons

```
AGGTCGGTGATGGGGTA---AGATGGGCGGCGCCGGCCT
AGGTCGGTGATGGGGTAGG-AGATGGGCGGCGCCGGCCT
AGGTCGGTGATGGGGTAGGTAGATGGGCGGCGCCGGCCT
AGGTCGGTGATGGGGTA---AGATGGGCGGCGCCGGCCT
AGGTCGGTGATGGGGTAGG-AGATGGGCGGCGCCGGCCT
AGGTCGGTGATGGGGTAGG-AGATGGGCGGCGCCGGCCT
AGGTCGGTGATGGG-------------CGGCGCCGGCCT
```

wildtype reference

```
AGGTCGGTGATGGGGTAGG-AGATGGGCGGCGCCGGCCT
```

t3 PAM

B433 T0 ENG1 contigs

```
T0 amplicon          GCGCCCCGGGAGA--------------------------------------------------------------------------------------------------------TGGGCGGCGCCGGCC
wildtype reference   GCGCCCCGGGAGAGGGGAGCGGCGTGGAGGCGCGGTTGAAGGGGAGGTGGAAGTTGGAGGCCTCGGAGAGGTAGGAGCGGGAGGCGAGCGCGGAGAGGTCGGTGATGGGGTAGGAGATGGGCGGCGCCGGCC
                                tA PAM                                                                                          t3 PAM
```

```
T0 amplicons         TCGGTGATGGG------------CGGCGCCGGC
                     TCGGTGATGGG------------CGGCGCCGGC
                     TCGGTGATGGG------------CGGCGCCGGC
wildtype reference   TCGGTGATGGGGTAGGAGATGGGCGGCGCCGGC
                                  t3 PAM
```

B435 T0 ENG1 contigs

```
T0 amplicons         TGGGGTAGGAGA--------------------GCGTCGAACGGCG
                     TGGGGTAGGAGATGGGCGGCGCCGGCCTGGAAGCGTCGAACGGCG
                     TGGGGTAGGAGATGGGCGGCGCCGGCCTGGAAGCGTCGAACGGCG
                     TGGGGTAGGAGATGGGCGGCGCCGGCCTGGAAGCGTCGAACGGCG
wildtype reference   TGGGGTAGGAGATGGGCGGCGCCGGCCTGGAAGCGTCGAACGGCG
                              t2 PAM
```

B436 T0 ENG1 contigs

```
T0 amplicons         CATGACCCTGC---------------------------------------------------------------------------------------CCTACCCCATCA
                     CATGACCCTGC---------------------------------------------------------------------------------------CCTACCCCATCA
                     CATGACCCTGC---------------------------------------------------------------------------------------CCTACCCCATCA
                     CATGACCCTGC---------------------------------------------------------------------------------------CCTACCCCATCA
                     CATGACCCTGCCCGCCGCGGACGACGCCGCCGCCTCCGAAGGCGAGCGGCGCGACTGGGAGCCGCCGTTCGACGCTTCCAGGCCGGCGCCGCCCTCT-CCTACCCCATCA
                     CATGACCCTGCCCGCCGCGGACGACGCCGCCGCCTCCGAAGGCGAGCGGCGCGACTGGGAGCCGCCGTTCGACGCTTCCAGGCCGGCGCCGCCCTCT-CCTACCCCATCA
wildtype reference   CATGACCCTGCCCGCCGCGGACGACGCCGCCGCCTCCGAAGGCGAGCGGCGCGACTGGGAGCCGCCGTTCGACGCTTCCAGGCCGGCGCCGCCCATCTCCTACCCCATCA
                                                                                                              t2 PAM
```

B438 T0 ENG1 contigs

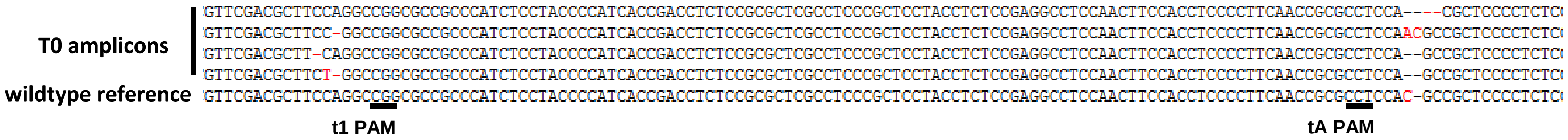

B439 T0 ENG1 contigs

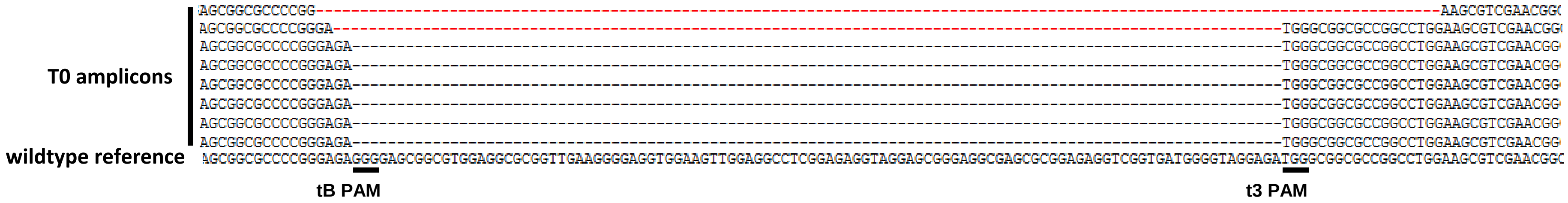

B440 T0 ENG1 contigs

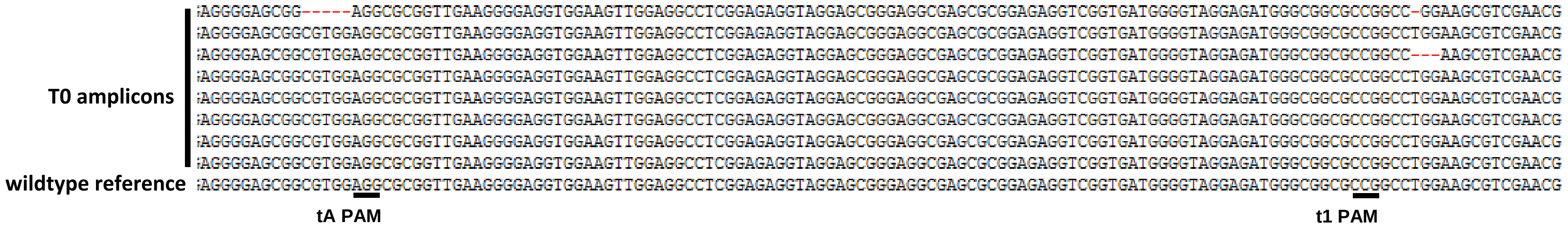

B442 T0 ENG1 contigs

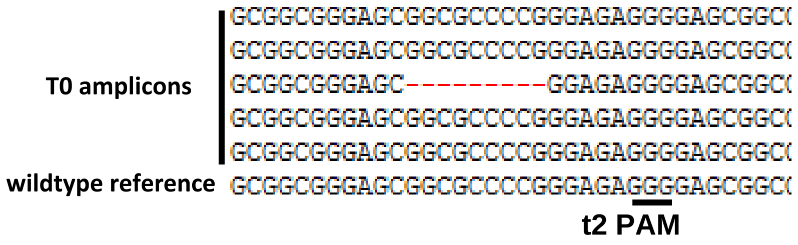

Supplement: Supplementary file 3 [file Data_Sheet_1.pdf]
